# Persistence of foliar applied and pre-storage seed-treated insecticides in rice and its processed products

**DOI:** 10.1038/s41598-024-53060-w

**Published:** 2024-01-29

**Authors:** A. Suganthi, R. Vigneshwari, N. Sathiah, M. Senthil Kumar, A. P. Sivamurugan, P. Thangachamy, S. S. Ilango, E. Madhu Sudhanan, P. Karthik, M. Shanthi

**Affiliations:** 1 Department of Agricultural Entomology, Tamil Nadu Agricultural University, Coimbatore, Tamil Nadu 641 003 India; 2 Department of Seed Science and Technology, Tamil Nadu Agricultural University, Coimbatore, Tamil Nadu 641 003 India; 3 Krishi Vigyan Kendra, Thiruvallur, Tamil Nadu Agricultural University, Thiruvallur, Tamil Nadu India; 4 Water Technology Centre, Tamil Nadu Agricultural University, Coimbatore, Tamil Nadu 641 003 India; 5 Centre for Plant Protection Studies, Tamil Nadu Agricultural University, Coimbatore, Tamil Nadu 641 003 India

**Keywords:** Plant sciences, Environmental sciences, Materials science

## Abstract

A field study was conducted to investigate the persistence of foliar-applied thiamethoxam 25% WG at a rate of 25 g ai ha^−1^ and chlorantraniliprole 18.5% SC at 30 g ai ha^−1^ in various parts of rice plants, including whole grain rice, brown rice, bran, husk, straw, and cooked rice. Liquid Chromatography-Mass spectrometry/Mass spectrometry was used for sample analysis. Chlorantraniliprole residues were found to persist in whole grains, bran, husk, and straw at the time of harvest, while thiamethoxam residue was not detected in harvested grains, processed products, or straw. The study concluded that foliar-applied chlorantraniliprole and thiamethoxam did not pose any dietary risk in cooked rice. In a pre-storage seed treatment study, thiamethoxam 30% FS at 3 mL kg^−1^ was evaluated against Angoumois grain moth infestation during storage. The seeds remained unharmed for nine months and exhibited significantly less moth damage (2.0%) even after twelve months of storage. Thiamethoxam residues persisted for more than one year in whole rice grain, brown rice, bran, and husk with seed treatment, with higher residue levels observed in bran and husk. Parboiling and cooking led to the degradation of thiamethoxam residues.

## Introduction

Rice, a major food staple globally and an extensively cultivated crop, is subject to significant pesticide applications through foliar sprays to manage economically important pests. Consequently, insecticide residues are present in rice grains and their products at harvest^1^, raising concerns about food safety. The neonicotinoid insecticide thiamethoxam is approved for foliar application in rice to combat stem borer, gall midge, leaf folder, white-backed plant hopper, brown plant hopper, green leaf hopper, and thrips^2^. It serves as a broad-spectrum insecticide and is also recommended as a seed treatment for safeguarding crops such as rice, maize and sunflower against early-stage insect pests. Thiamethoxam's effectiveness as a seed treatment chemical has been reported in maize, cotton, and oilseed rape^3^. Another widely used insecticide in rice crops is chlorantraniliprole^4^, a diamide insecticide that binds to ryanodine receptors (RyR) in insect muscle, disrupting normal muscle contraction. Given the escalating concerns about food safety, the persistence of insecticides applied to rice crops and their residues becomes crucial, considering that rice is a staple food for the majority of the world’s population.

With a global production of 756 million tonnes, rice stands as the world’s third-most-produced agricultural crop. As the global population continues to grow, rice remains a crucial staple food^5^. The demand for milled and parboiled rice from countries such as India, Pakistan, and Thailand is on the rise, with countries like the U.S. and Europe increasing their imports. In major rice-producing nations, 8 to 26% of rice is lost due to post-harvest issues and inadequate infrastructure. Grains are stored by governments, farmers for personal consumption or seed purposes, and traders for potential financial gains by speculating on increased commodity prices in the future.

Quality seeds play a vital role in realizing the yield potential of any crop species. India, the second-largest rice producer globally, contributes 21.5% to world rice production, covering 24% of the country's gross cropped area. Production and distribution of high-quality seeds are key objectives for all seed producers. However, the seed production system prohibits bulk storage of seed lots. Certified seed material must be individually packed in jute or cloth bags and stored as such until sowing or the regulatory period of nine months. Consequently, drying, reprocessing, and treating seed lots midway through the validity period become challenging. Protecting seeds from insect damage during this crucial period is highly challenging. Seed material deterioration is a biological process that cannot be stopped or reversed but can be controlled by storing seeds at the proper moisture content and temperature^6^. The Angoumois grain moth, *Sitotroga cerealella* (Olivier), or the rice grain moth, is recognized as one of the most destructive internal feeders in stored rice grains^7^. It accounts for over 40% of total losses in stored grain in some areas^8,9^.

In developing countries, approximately 50 to 60% of grains are stored in traditional structures at the household and farm level^10^. The use of pesticides for seed treatment is a crucial management practice against storage pests, ensuring the seed's vitality until it is used for sowing. Given the economic importance of quality seeds, thiamethoxam 30 FS formulation, recommended as a seed dresser^11^, was selected for the pre-storage treatment of rice seeds and studied for its bio-efficacy and residues.

Pesticide residues in rice grains and processed foods raise concerns and pose serious health hazards for humans. The risk associated with bulk-stored seeds treated with insecticides requires attention, considering the potential conversion of the seed for grain purposes or as animal feed in unexpected situations. Published data on the persistence of pre-storage seed-treated chemicals in rice are scarce. Studies from around the world have reported both the presence and absence of pesticide residues in rice following application to the aerial parts or after seed treatment^12,13^. Understanding the persistence of seed protectants is crucial for assessing the efficacy of insecticides over time, as residues on freshly treated seeds and long-stored seeds may vary.

The processing of rice grains significantly influences the level of residues. Whole rice grains undergo processing to remove the husk and a specific level of bran from the rice endosperm while minimizing grain breakage. The rice obtained after husk removal is termed brown rice, and the extracted bran, rich in nutrients, is primarily used for oil production and as animal feed.

Against this backdrop, our research aimed to accomplish three objectives:

(1) Investigate the persistence of foliar-applied thiamethoxam and chlorantraniliprole in rice crops and their products. (2) Evaluate the effectiveness of thiamethoxam in safeguarding stored rice seeds from angoumois grain moth infestation and assess seed quality characteristics post pre-storage treatment with thiamethoxam. (3) Examine the persistence of insecticide residues in stored rice seeds, brown rice, husk, bran, parboiled and cooked products.

A field study was undertaken to examine the persistence of foliar-applied chlorantraniliprole and thiamethoxam in rice crops. Additionally, a laboratory storage seed treatment study was conducted to assess the persistence of thiamethoxam residues in treated rice seeds and their processed products over a 12-month storage period, correlating the insecticide's effectiveness in controlling grain moth pests.

## Materials and methods

### Reagents and chemicals

Certified reference materials of thiamethoxam (99.70% purity) and chlorantraniliprole (97.63% purity) were purchased from Sigma Aldrich (India). HPLC-grade acetone, MS-grade acetonitrile and formic acid, magnesium sulfate (MgSO_4_), and anhydrous sodium chloride (NaCl) analytical-reagent grade were purchased from Merck India Ltd. (Merck, Germany). Primary Secondary Amine (PSA) (Bondesil 40 μm) and Graphitized Carbon Black (GCB) were sourced from Agilent Technologies, USA. To prepare standard stock solutions (at 400 μg mL^−1^), accurate amounts of reference material for thiamethoxam and chlorantraniliprole were dissolved in acetonitrile and stored at 4 °C. Working standard solutions were then created in acetonitrile through suitable dilutions of aliquots from the original stock solution as needed. Ultra-pure water, collected from the Merck Millipore water unit (Precision Scientific Co, Coimbatore, India), was used in the experiments. Thiamethoxam 25 WG, thiamethoxam 30 FS, and chlorantraniliprole 18.5 SC formulations were purchased from a retail pesticide outlet in Coimbatore, India.

### Field trial

Thiamethoxam and chlorantraniliprole, two pesticides designed for insect pest control in rice crops, were selected for foliar application. A field experiment was conducted in Coimbatore, Tamil Nadu, India, during the 2020–2021 season, focusing on the dissipation pattern and persistence of residues of thiamethoxam 25 WG (applied at 25 and 50 g ai ha^−1^) and chlorantraniliprole 18.5 SC (applied at 30 and 60 g ai ha^−1^) in rice and its processed products. In the residue studies, double the recommended dose was incorporated to account for worst-case scenarios involving excessive application by rice farmers. A spray fluid volume of 500 L ha^−1^ was utilized, with thiamethoxam dilutions of 0.2 and 0.4 g L^−1^ and chlorantraniliprole dilutions of 0.3 and 0.6 ml L^−1^, applied using a high-volume knapsack sprayer.

Control plots, without the application of the aforementioned insecticides, were maintained separately. The experiments were arranged in a Randomized Complete Block Design with treatments replicated thrice. Seedlings were transplanted at a spacing of 20 × 15 cm, and all necessary agronomic practices were conducted following Tamil Nadu Agricultural University (TNAU) recommendations, except for pesticide spray^14^. The experimental research and field studies on cultivated rice plants, including the collection of plant material, adhered to relevant institutional guidelines.

The initial spraying was done 25 days after transplanting, followed by the second and third sprays at 10-day intervals. Leaf samples were collected at various time points: 0 (within 2 h of the last application), 3, 7, 14, and 30 days after the third spraying. Additionally, samples of rice grains and straw were collected at the time of harvest for residue analysis. The harvested grains, following the processing procedures outlined in section “Processing of rice grains”, were analyzed, along with straw, to detect residues of foliar-applied thiamethoxam and chlorantraniliprole.

### Pre-storage seed treatment

In another experiment, the seeds of rice ADT 45 cultivar obtained from the Department of Agronomy, TNAU, were dried to a moisture level of 11 to 12%. Healthy seeds were subjected to treatment with thiamethoxam 30 FS at a rate of 3 mL kg^−1^ seeds. The recommended insecticide dose per kilogram of seed was mixed with distilled water to achieve a volume of 20 mL. This insecticidal solution was then combined with the seeds in a zip-lock plastic bag, manually shaken for one minute to ensure uniform coverage, and subsequently placed in plastic trays for shade-drying for one hour. The treated seeds, packed in 2 kg gunny bags, were stacked separately and stored for 12 months. Control samples, subjected to the same storage conditions, were stored separately. The entire experiment was conducted under ambient storage conditions (33 ± 2 °C and 57% RH) at the Department of Seed Science and Technology, TNAU, in a completely randomized design with three replications. Monthly evaluations were performed on the treated seeds, including assessments for insect damage (%), germination, seedling vigor, electrical conductivity, and insecticide residues.

The efficacy of thiamethoxam as a seed treatment chemical was assessed using a visual damage scale, measuring the percentage of grains exhibiting pinhole damage symptoms caused by the rice moth^15^ (Utono, 2013). Observations were recorded for 100 treated seeds and in the control treatment, and damage symptoms were expressed as a percentage. The germination test involved 100 seeds, conducted by the paper method in four replicates, at a temperature of 25 ± 1 °C and a relative humidity of 95 ± 2%. Germination percentage was calculated based on normal seedling count on the 14th day^16^, and the seedling vigor index was computed using the formula suggested by Baki and Anderson^17^, expressed as a whole number:

$${\text{Vigor}}\;{\text{Index}} = {\text{Germination}}\;{\text{percent}} \times {\text{Total seedling length in cm}}.$$

Electrical conductivity was measured using an electrical conductivity bridge with a cell constant of 1.0. Seed leachate obtained from 50 pre-washed seeds soaked in 50 ml of deionized water for 16 h at room temperature was used for the measurement^18^. Stored rice seed samples were drawn monthly up to twelve months of storage for processing and residue analysis, as outlined in sections “Processing of rice grains” and “Residue analysis”, respectively.

### Processing of rice grains

Grains harvested from the foliar-sprayed field experiment and seed samples (referred to as grains hereafter) obtained from pre-storage treatment underwent processing for value addition. At each processing step, the products were collected and subjected to residue analysis. Residues were analyzed in raw whole grains and their processed forms, including parboiled whole grain, cooked raw rice, cooked parboiled rice, husk, and bran from both raw whole grain and parboiled whole grain.

To ensure uniformity, the moisture content of stored rice grains was maintained at 12 ± 1% before processing. The parboiling process involved soaking the grains in excess water for 6 h, followed by steam heating at 100 °C with husk for 20 min. The grains were then drained and dried in medium shade. This parboiling process not only facilitates easy dehusking but also reduces losses during milling^19^.

Dehusking of both raw and parboiled whole grains was performed by hand pounding using a mortar and pestle at room temperature (33 ± 2 °C). Thorough washing of the mortar was done each time to prevent cross-contamination between samples. After pounding, grains were aspirated to remove the husk. The processed grain fraction was then sieved through a 30-mesh sieve (595 μ particle size) to eliminate the bran. For cooking raw and parboiled rice (after husk and bran removal), a 1:2 ratio of rice to water was used. Water was boiled first, and then rice was added, allowed to cook for 20 min until all the liquid was absorbed.

### Residue analysis

An efficient analytical method was standardized by modifying the QuEChERS method^20,21^. This method was validated using LC–MS/MS to detect and quantify: (1) Foliar-applied thiamethoxam and chlorantraniliprole residues in samples of rice leaves, whole grain, straw, husk, bran, parboiled grain, cooked raw and parboiled rice from the field trial. (2) Residues of thiamethoxam in pre-storage treated seeds and its processed products.

#### Instrument conditions

The study employed a liquid chromatographic instrument (Make: Waters, USA; Model: Alliance 2695) equipped with a quaternary pump, autosampler, inbuilt degasser, and a suitable Waters XTerra-C18 column (5 µm; 4.8 × 250 mm). This instrument was coupled with Acquity TQD mass spectrometry featuring an ESI interface. Data acquisition during sample analysis and runs utilized Masslynx software version 4.1, 2005 (Waters, Milford, MA, USA). For the mobile phase, acetonitrile: water acidified with 0.1% formic acid (30:70, v/v) was employed at a flow rate of 0.5 mL min^−1^, resulting in the elution of analytes within eight minutes of the run time.

MS/MS conditions were optimized, and parent and daughter ions were identified by directly infusing individual standard solutions prepared in acetonitrile/water (50/50, v/v). Two different m/z transitions were chosen in the ESI-positive mode. Specifically, transitions m/z 292.11 > 210.92 and 131.91, and 482.13 > 283.87 and 119.91 were used for quantification and confirmation of thiamethoxam and chlorantraniliprole residues, respectively. Optimal cone and collision voltages were set at 20 and 22, and 10; 24 and 12; 62 for the daughter ions of thiamethoxam and chlorantraniliprole, respectively. The ion source and desolvation gas temperatures were maintained at 150 and 500 °C, respectively. Desolvation gas (nitrogen), cone gas (nitrogen), and collision gas (argon) flow rates were set at 1100 L h^−1^, 80 L h^−1^, and 0.18 mL min^−1^, respectively.

For the final determination of residues, a sample volume of 10 µL was injected by an autosampler into the LC–MS/MS. The final quantification was worked out using the following formula with the parameters from the chromatogram.

$${\text{Residues}}\;({\text{mg}}\;{\text{g}}^{ - 1} ) = \frac{{{\text{As}}}}{{{\text{Astd}}}} \times \frac{{{\text{Wstd}}}}{{{\text{Ws}}}} \times {\text{Vs}}$$

As—Peak area of the sample; Astd—Peak area of the standard; Wstd—Weight of the standard in µg mL^−1^;Ws—Weight of the sample in g; Vs—Volume of the sample (final extract in mL).

#### Sample preparation methods

A representative sample of 2 g of rice bran/husk was accurately weighed into a 50 mL centrifuge tube and mixed with 10 mL water, allowing it to stand for 20 min. The sample was then mixed with 20 mL acetonitrile, vortexed for 2 min, and supplemented with approximately 4 g of anhydrous MgSO_4_ and 1 g of NaCl. The mixture was shaken thoroughly using a vortexer, followed by centrifugation at 6000 rpm for 10 min.

After centrifugation, a 6 mL aliquot of the supernatant was transferred into a 15 mL centrifuge tube containing 100 mg PSA and 600 mg anhydrous MgSO_4_. The mixture was vortexed for one minute and then centrifuged for 10 min at 3000 rpm. Subsequently, a 4 mL aliquot of the supernatant was transferred into a TurboVap tube and dried under a stream of nitrogen in a TurboVapLV (Caliper Life Sciences, Hopkinton, USA) at 40 °C. The dried extract was then redissolved in 1 mL of acetonitrile and transferred into a 1.5 mL glass vial for LC–MS/MS analysis. For the analysis of rice whole grain, brown rice, and cooked rice, a 10 g sample was taken and extracted with 20 mL acetonitrile, followed by the same processing steps as described above.

Leaf and straw sample analysis involved taking a 2 g coarsely ground sample, which was then mixed with 5 mL of water and extracted using 10 mL of acetonitrile. During the clean-up step, 10 mg of Graphitized Carbon Black (GCB) was used in conjunction with 100 mg of PSA and 600 mg of anhydrous MgSO_4_. A 4 mL aliquot of the supernatant was concentrated in the TurboVap LV at 40 °C to near dryness. The resulting extract was redissolved in 1 mL of acetonitrile and transferred into a 1.5 mL glass vial for LC–MS/MS analysis.

#### Method validation

Paddy grains and straw samples collected from an organic field were utilized for method validation studies^22^, detailed in the Supplementary Material and Fig. S1.

Method validation for residue analysis of thiamethoxam and chlorantraniliprole was conducted following the SANTE (2019) guidelines. Five attributes of the extraction and analysis methods were validated: Linearity, accuracy, precision (% RSD), the limit of detection (LOD), and limit of quantification (LOQ). The recovery (accuracy) and precision study involved spiked rice matrices at five concentration levels, following the standardized method using LC–MS/MS.

### Data analysis

The pre-storage seed treatment experiment was designed as a factorial completely randomized design (f-CRD). To assess the significant effects (P < 0.05) of thiamethoxam on seed storage parameters and residue, analysis of variance (ANOVA) was conducted, followed by Duncan’s multiple range test, utilizing SPSS Statistics for Windows, Version 23.0 (IBM SPSS, Armonk, NY: IBM Corp.). For statistical analysis of seed quality data, percentage values were transformed into an arc sine value to ensure homogeneity of variance. Critical differences (CD) were calculated at a 5% probability level. The data underwent rigorous testing for statistical significance.

Residue analysis experiments were conducted in triplicate, and the average values were reported. Half-life and pre-harvest intervals^23^ were calculated using the following mathematical formula:

$${\text{Half-life}} ({\text{t}}_{1/2})=\frac{{\log }2}{|{\text{b}}|}$$

where b is the slope of the regression line.

The pre-harvest interval was calculated by using the formula,

$${\text{T}}_{{\rm tol}} ({\text{Days}})=\frac{[{\mathrm{a}}-{\text{Log}}\left({\text{MRL}}\right)]}{|{\text{b}}|}$$

where T_tol_ is the minimum time (days) taken by the pesticide to reach below the tolerance limit; a = Log of initial deposit of residue (μg g^−1^); b = Slope of the regression line.

## Results and discussion

The developed method was employed to determine residues of thiamethoxam and chlorantraniliprole in rice plants and their products. Following foliar application, the initial deposit of thiamethoxam in rice leaves was 0.30 and 0.98 μg g^−1^ for recommended and double the recommended dose, respectively, while chlorantraniliprole deposits were 1.11 and 2.25 μg g^−1^ (Table 1). The initial thiamethoxam deposit in leaves was below the MRL of 2 mg kg^−1^ fixed for straw^24^, and the residues dissipated below quantifiable limits within 7 days. No residues of thiamethoxam were detected in the harvested rice grains, bran, husk, and straw, indicating its rapid dissipation. Telo et al.^25^ also observed an 88% reduction in the initial concentration of thiamethoxam in rice plants after 15 days of application, with no residues found in rice grains despite processing. The results suggest that applying thiamethoxam 25 to 50 days after transplanting will not leave any residues in rice grains, their processed products, and straw.

**Table 1:** Persistence and dissipation of foliar applied thiamethoxam and chlorantraniliprole in /on rice leaves, harvest time rice and its produces.

*Reesidues in Rice leaves* (Days after spraying)	Residues (μg g^−1^)			
	Thiamethoxam 25 WG @ 25 g ai ha^−1^	Thiamethoxam 25 WG @ 50 g ai ha^−1^	Chlorantraniliprole 18.5 SC @ 30 g ai ha^−1^	Chlorantraniliprole18.5 SC @ 60 g ai ha^−1^
0	0.30 ± 0.03	0.98 ± 0.04	1.11 ± 0.10	2.25 ± 0.03
3	0.12 ± 0.01	0.25 ± 0.01	0.86 ± 0.03	1.74 ± 0.02
7	BQL	BQL	0.54 ± 0.001	1.56 ± 0.01
14	–	–	0.41 ± 0.02	1.40 ± 0.04
30	–	–	0.40 ± 0.001	0.86 ± 0.01
*Residues in Harvest time rice and produces*				
Paddy whole grain	ND	ND	BQL	0.03 ± 0.02
Raw rice			ND	ND
Husk			0.14 ± 0.05	0.33 ± 0.05
Bran			0.17 ± 0.03	0.46 ± 0.06
Parboiled rice			ND	ND
Bran—Parboiled grain			BQL	0.04 ± 0.004
Husk—Parboiled grain			BQL	0.047 ± 0.002
Straw			0.89 ± 0.05	1.32 ± 0.03
Raw rice – Cooked			ND	ND
Parboiled rice -Cooked			ND	ND

BQL—Below Quantification Level (0.025 μg g^−1^); ND—not detected.

In contrast, chlorantraniliprole residues demonstrated prolonged persistence, exceeding 30 days in leaves after application, with a calculated half-life ranging from 14 to 17 days. However, the residue levels remained below the Maximum Residue Limit (MRL) of 30 mg kg^−1^ set by the Codex Alimentarius Commission for chlorantraniliprole residues in rice straw^24^. Analysis of harvest-time rice samples from the field trial disclosed the presence of chlorantraniliprole residues in various components, including rice whole grain, husk, bran, and straw. The pre-harvest interval set for rice straw was 70 days. Interestingly, no residues were detected in rice whole grain or polished rice grain at the recommended dose, while detectable residues (0.03 μg g^−1^) were observed at higher doses, still well below the MRL of 0.4 mg kg^−1^, indicating a potential risk of residues in grains if the dosage exceeds recommendations. Given its non-polar nature, chlorantraniliprole exhibited higher persistence and exhibited a greater affinity for rice bran, which is rich in fat. The prospect of removing chlorantraniliprole residues through parboiling is remote, given its high melting point of 208 to 210 °C. The absence of residues in parboiled rice is likely attributed to the removal of bran and husk during the parboiling process. The moderate water solubility of chlorantraniliprole (0.880 mgL^−1^)^26^ and its increased absorption in lipophilic grain components contribute to its persistence in the bran and husk of parboiled rice. As residue levels in bran surpass those in grain, this underscores the necessity for establishing regulatory limits for bran used in animal feed.

In contrast to our current findings, Bhardwaj et al.^4^ reported straw samples at harvest time that were free of residues after a single application of chlorantraniliprole. The persistence of residues, however, varies based on the duration of the crop and the number of insecticide applications. Notably, the drying of rice leaves resulted in higher concentrations of residues in straw compared to green leaves, primarily due to moisture loss.

Several researchers have investigated the presence of pesticide residues in rice grains. The application of thiamethoxam and lambda-cyhalothrin at both the recommended and double-recommended rates to rice crops yielded grains devoid of any residues^13^. Conversely, in a farmgate sample, thiamethoxam residue was reported in the whole rice grain^21^. Chlorantraniliprole residues (0.027 mg kg^−1^) were detected in brown rice grains following the application of chlorantraniliprole, both during and at the end of the rice crop cycle in China^12^. In a similar study, residues of both thiamethoxam and chlorantraniliprole were reported in processed rice products^25^ where pesticide application occurred after flowering. The concentration of insecticide residues in rice grain may vary depending on factors such as the part of the grain (hull, bran, whole grain, or polished grain), the chemical nature of the insecticide, and the stage of the crop during application.

### Seed quality and residues of seed treated thiamethoxam:

The results of the study revealed significant differences in seed quality parameters, including germination, vigor index, insect damage, and electrical conductivity, between control seeds and those treated with thiamethoxam (30 FS @ 3 mL kg^−1^) during storage, as shown in Table 2. After 12 months of storage, the treated seeds exhibited an impressive 82% germination rate, while only 73% germination was observed in the untreated seeds^27^. Both untreated and treated seeds maintained a stable germination percentage until three months, after which it gradually decreased with the extension of the storage period.

**Table 2:** Influence of thiamethoxam seed treatment and storage period on rice seed germination, vigor, insect damage and electrical conductivity.

Storage period (Months)*	Control					Thiamethoxam 30 FS @ 3 mL kg^−1^				
	Germination (%)	Seedling length (cm)	Vigour Index	Insect damage (%)	EC (µScm^−1^ g^−1^)	Germination (%)	Seedling length (cm)	Vigour Index	Insect damage (%)	EC (µScm^−1^ g^−1^)
P_0_	92 (73.57)	25.4	2337	0	33.12	92 (73.57)	25.1	2309	0	33.57
P_1_	92 (73.57)	25.1	2309	0	35.64	92 (73.57)	24.9	2291	0	34.25
P_2_	91 (72.54)	24.8	2257	0	37.81	91 (72.54)	24.6	2239	0	36.09
P_3_	89 (70.63)	24.5	2181	2.4 (8.91)	40.25	91 (72.54)	24.8	2257	0	40.11
P_4_	87 (68.87)	24.0	2088	3.2 (10.30)	43.29	90 (71.57)	24.5	2205	0	42.31
P_5_	86 (68.03)	24.1	2073	4.6 (12.38)	45.73	89 (70.63)	24.6	2189	0	44.87
P_6_	85 (67.21)	23.8	2023	6.5 (14.77)	48.05	89 (70.63)	24.4	2172	0	47.48
P_7_	82 (64.90)	23.4	1919	8.4 (16.85)	53.86	87 (68.87)	23.8	2071	0	52.67
P_8_	81 (64.16)	23.1	1871	10.5 (18.91)	56.78	86 (68.03)	24.1	2073	0	55.33
P_9_	80 (63.43)	22.8	1824	13.8 (21.81)	62.14	85 (67.21)	23.7	2015	0	60.05
P_10_	77 (61.34)	22.5	1733	15.4 (23.11)	66.48	85 (67.21)	22.5	1913	1.3 (6.55)	63.74
P_11_	75 (60.00)	22.2	1665	17.2 (24.50)	69.61	84 (66.42)	22.2	1865	1.7 (7.49)	66.32
P_12_	73 (58.69)	21.7	1584	19.5 (26.21)	71.53	82 (64.90)	22.3	1829	2.0 (8.13)	68.96
Mean	84 (66.42)	23.6	1989	7.8 (16.22)	51.10	88 (69.73)	24.0	2110	0.4 (3.63)	49.67
SEd for treatment (T)		0.4	0.12	13.14	0.23	0.39				
SEd for period (P)		1.1	0.30	33.51	0.58	1.00				
SEd for T × P		1.5	0.42	47.38	0.82	1.41				
CD (P = 0.05)for treatment (T)		0.8	0.23	26.16	0.45	0.78				
CD (P = 0.05)for period (P)		2.1	0.60	66.71	1.15	1.99				
CD (P = 0.05)forT × P		NS	NS	94.33	1.63	NS				

*P_0–12_—Month 0 to 12.

Furthermore, the vigor index value was notably higher in seeds treated with thiamethoxam compared to control seeds. This difference was attributed to the substantial infestation and depletion of food reserves in untreated seeds caused by insect pests. The decline in germination percentage and vigor index was more pronounced in untreated seeds, and this was associated with damage by Angumois grain moth in the control seeds, ranging from 2.4 to 19.5%. Interestingly, the treated seeds remained unharmed until nine months and displayed significantly less grain moth damage (2.0%) even after twelve months of storage.

In line with these findings, Arthur et al.^28^ reported a high level of control (90 to 100%) against *Rhyzopertha dominica* (Fabricius) and *Sitophilus oryzae* (Linnaeus) in wheat seeds treated with thiamethoxam at concentrations ranging from 1 to 4 ppm. These results underscore the efficacy of thiamethoxam in preserving seed quality and protecting against insect damage during storage.

Thiamethoxam-treated rice seeds exhibited lower electrical conductivity, measuring at 49.67 μS cm^−1^, which can be attributed to reduced insect damage in the treated seeds. Electrical conductivity tests in seeds serve as an indirect measure of cell membrane damage resulting from seed deterioration^29^. Insects' impact on grains typically leads to an increase in electrical conductivity as the outer wall ruptures and perforations occur in the grain's tegument^30^.

Beyond its protective role, thiamethoxam was reported to possess bio-activator properties^31^. When used for seed treatment, it increased the expression of seed vigor, dry matter accumulation, root length, and photosynthetic processes, enhancing seed performance in various crops such as rice^32^, cotton^33^, and soybean^31^. The effectiveness of seed treatment chemicals may vary depending on factors such as the type of formulation used, treatment conditions, storage duration, environmental storage conditions, and the specific crop involved.

The current results indicate that, when treated with thiamethoxam, rice seed germination remained unaffected by the storage period up to one year. Pre-storage seed treatment with thiamethoxam has also been reported to enhance seed germination and vigor in bean seeds^34^ and improve the physiological quality of pumpkin seeds^35^. However, it's worth noting that Dan et al.^31^ reported a reduction in the seed vigor of thiamethoxam-treated soybeans. These findings highlight the importance of considering specific crop and treatment variations in assessing the impact of thiamethoxam on seed quality.

**Figure 1:**
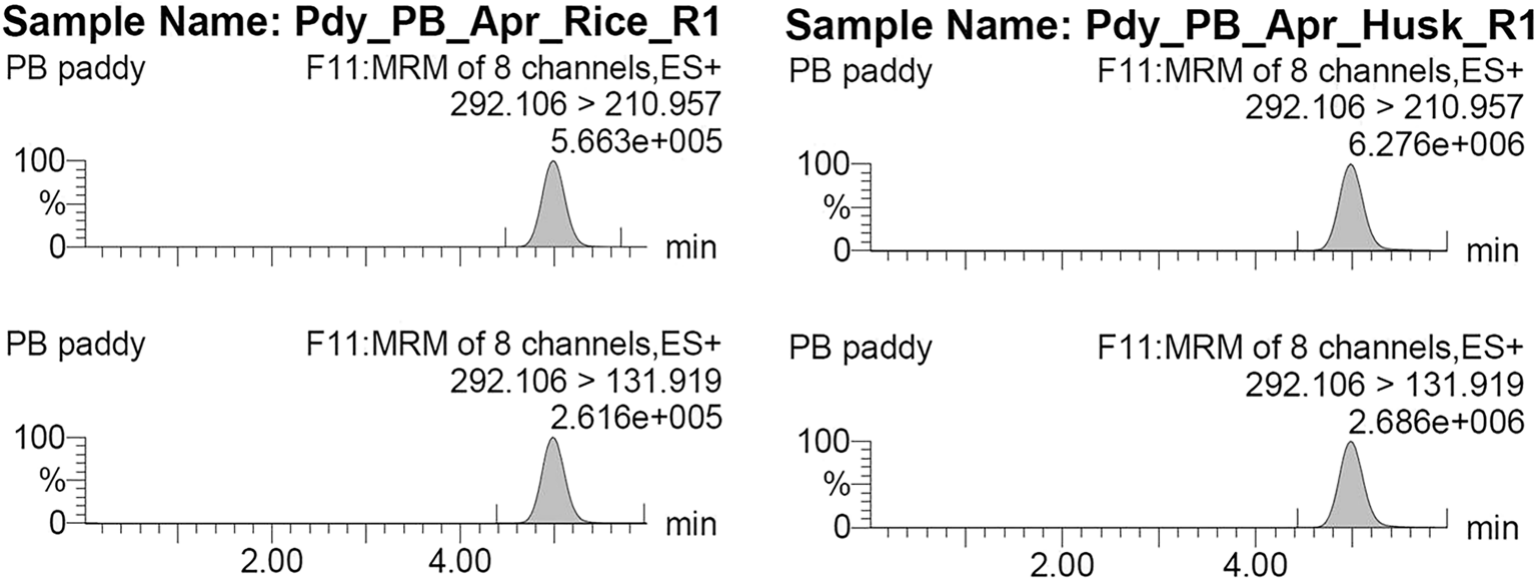
Chromatogram of thiamethoxam residues in par boiled rice and husk (one month after pre-storage treatment).

In our seed treatment study, thiamethoxam residues exhibited a slow decline and were still detectable in various components of rice, including whole grains (seeds), husk, bran, and cooked rice, even after twelve months (Table 3) (Fig. 1). This prolonged persistence of thiamethoxam as a seed treatment chemical likely contributes to the significantly lower levels of insect damage observed even after a year of storage.

**Table 3:** Persistence of seed treated thiamethoxam 30 FS @ 3 mL kg^−1^ in stored rice grains and its products.

Matrix	Residue across different treatment periods (μg g^−1^)												
	Feb. 2021	Mar. 2021	Apr. 2021	May 2021	June2021	July 2021	Aug2021	Sept 2021	Oct. 2021	Nov. 2021	Dec.2021	Jan.2022	Feb. 2022
*Raw grain*													
Grain	1632.01 ± 5.28	634.05 ± 0.62	599.77 ± 0.21	559.34 ± 0.13	537.31 ± 0.06	545.90 ± 0.65	425.59 ± 0.13	400.66 ± 0.014	313.70 ± 0.76	108.67 ± 3.38	61.15 ± 1.11	50.29 ± 0.72	24.61 ± 0.24
Rice	614.48 ± 8.60	82.47 ± 0.02	85.86 ± 0.11	83.17 ± 0.02	83.92 ± 0.02	75.66 ± 0.01	74.84 ± 0.02	76.80 ± 0.04	58.34 ± 0.18	52.81 ± 1.07	36.56 ± 0.58	23.52 ± 0.17	12.05 ± 0.28
Bran	1447.08 ± 71.07	973.44 ± 2.05	698.09 ± 2.34	459.38 ± 1.79	395.42 ± 2.06	389.48 ± 1.53	305.28 ± 0.90	233.66 ± 0.71	299.00 ± 2.84	246.78 ± 14.39	196.01 ± 16.06	162.70 ± 4.40	30.83 ± 0.46
Husk	1285.32 ± 27.59	867.76 ± 6.22	607.97 ± 3.11	396.71 ± 6.12	360.85 ± 1.96	332.97 ± 0.12	293.47 ± .0.60	253.61 ± 0.51	252.22 ± 0.19	204.98 ± 6.87	153.73 ± 6.26	150.34 ± 1.74	23.85 ± 0.23
CD (0.05%) SEd	P 25.53** 12.87	T 14.16 7.14	P × T 51.07** 25.75										
*Parboiled grain*													
Grain	756.0 ± 3.60	376.01 ± 8.98	332.85 ± 1.19	269.82 ± 3.47	119.06 ± 3.95	110.50 ± 2.37	79.55 ± 0.22	11.23 ± 0.46	6.65 ± 0.02	1.67 ± 0.01	0.82 ± 0.04	0.67 ± 0.02	1.39 ± 0.05
Rice	126.68 ± 38.22	62.98 ± 8.22	46.38 ± 1.14	38.13 ± 0.93	16.62 ± 0.93	13.92 ± 1.51	7.66 ± 0.31	1.41 ± 0.24	0.93 ± 0.02	0.43 ± 0.01	0.45 ± 0.10	0.34 ± 0.00	0.16 ± 0.04
Bran	1052.36 ± 239.86	338.45 ± 1.24	303.36 ± 5.73	239.88 ± 6.10	102.25 ± 6.10	97.52 ± 3.36	62.49 ± 11.14	52.68 ± 3.31	30.34 ± 0.67	13.93 ± 0.11	6.18 ± 0.24	4.73 ± 0.14	3.19 ± 0.07
Husk	616.58 ± 7.91	593.62 ± 9.87	537.20 ± 5.04	437.14 ± 3.93	189.17 ± 3.92	179.44 ± 5.66	96.01 ± 3.21	60.05 ± 5.69	53.72 ± 0.50	24.31 ± 0.08	11.15 ± 0.11	8.87 ± 0.34	3.02 ± 0.34
CD (0.05%) SEd	P 27.55** 13.89	T 15.28** 7.7	P × T 55.11** 27.80										
*Cooked rice*													
Cooked Raw rice	8.26 ± 0.35	5.71 ± 0.00	5.46 ± 0.02	4.04 ± 0.09	3.44 ± 1.11	2.32 ± 0.04	2.67 ± 1.05	2.85 ± 0.01	2.43 ± 1.01	1.05 ± 0.02	0.78 ± 0.01	0.72 ± 0.02	0.28 ± 0.04
Cooked Parboiled rice	1.09 ± 0.06	1.20 ± 0.10	0.84 ± 0.01	1.06 ± 0.09	0.86 ± 0.00	0.54 ± 0.09	0.53 ± 0.07	0.47 ± 0.04	0.23 ± 0.01	0.28 ± 0.01	0.27 ± 0.01	0.18 ± 0.01	0.11 ± 0.01
CD (0.05%) SEd	P 0.397** 0.198	T 0.155** 0.077	P × T 0.562** 0.28										

P—Storage period, T—Product matrix.

Among raw whole grain products, thiamethoxam residues were found to be higher in bran compared to husk and rice, whereas in parboiled grains, residues followed the order of husk > bran > rice whole grain > rice. Bran, situated between the rice grain and husk, is removed during processing. It possesses unique properties distinct from those of the grain and husk, containing 13 to 23% fat^36^. Lipophilic insecticides, such as thiamethoxam, may translocate to the bran rich in triglycerides. Consistent with this, a prior study^25^ also reported more pesticide residues in rice hull and bran samples. As stored rice seeds age, bran, rich in lipase, hydrolyzes bran oil to free fatty acid and glycerol. Free fatty acid content in bran was reported to increase from 4.36 to 24.70% after four weeks of storage^37^. This could be a factor contributing to the higher persistence of thiamethoxam residues in bran during prolonged storage. Even after twelve months of storing treated seeds, thiamethoxam residue exceeded the Japanese MRL of 0.3 μg g^−1^^38^ in whole rice grain and 0.02 μg g^−1^ in rice, as per the Food Safety and Standards Authority of India^39^. Over the 12-month storage period, residues degraded by 96.17%, 96.92%, 88.76%, and 88.30%, respectively, in raw whole grain, rice, bran, and husk.

Parboiling of grains resulted in the removal of residues to the extent of 40.70% to 98.66%, 23.64% to 99.18%, 27.28% to 97.10%, and 11.64% to 94.10% in whole grain, rice, bran, and husk, respectively. The initial residues in cooked raw rice and parboiled rice were 8.26 μg g^−1^ and 1.09 μg g^−1^, respectively. Cooking further reduced residues by 93.08% to 98.86% and 31.25% to 99.14% in raw rice and parboiled rice, respectively.

The decrease in thiamethoxam residues observed after parboiling and cooking in rice can be attributed to thermal degradation. Boiling or cooking processes can induce hydrolysis, volatilization, or chemical degradation, leading to a reduction in residue levels. The efficacy of this degradation process is also influenced by the solubility of the insecticide in water. Thiamethoxam has a water solubility of 4.1 × 10^3^ mg L^−1^ at 25 °C, and its thermal decomposition initiates at around 147 °C. During the soaking and cooking stages of parboiling, there is an increased likelihood of removing water-soluble thiamethoxam residues present on the grain surface, germ, and pericarp.

Various domestic and industrial processes, such as washing, parboiling, and cooking, have been reported to reduce residue levels in foods treated with pesticides^40,41^. Consistent with this, Telo et al.^25^ reported higher quantities of thiamethoxam and chlorantraniliprole residues in rice hull and bran compared to polished rice grains. The persistence of pesticide residues in food commodities is influenced by factors such as their chemical properties, time and method of application, environmental conditions, and adsorption and degradation behaviors^42,43^.

## Conclusion

The foliar application of chlorantraniliprole resulted in residues in bran, husk, and rice straw, while thiamethoxam residues were below the quantifiable limit. This highlights the importance of implementing a proper pre-harvest interval after chlorantraniliprole application to ensure food safety. In contrast, pre-storage seed treatment with thiamethoxam at the studied use level showed significantly less damage from Angumois grain moth but still contained residues even after 12 months of storage. Given that rice is a staple food and a valuable natural resource, the study underscores the potential of converting treated seeds into grain, even in unexpected situations, when using reduced-risk insecticides.

Despite these promising findings, further specific research is necessary to establish the appropriate chemical, formulation, and dose, aligning with applicable laws and regulations for chemical seed treatment to minimize risks. Notably, there are currently no guidelines for disposing of surplus or large quantities of pesticide-treated seeds, which pose environmental hazards and fall outside existing regulations. In cases where treated seeds lose viability and carry unacceptable risks for human consumption, a method of disposal is not yet established. Thus, there is a pressing need to develop a strategy for the pre-storage treatment of seed grains by seed producers. Studies such as this serve as a foundational step for identifying suitable chemicals for storage treatment and provide valuable insights for policymakers and regulatory authorities in monitoring treated seeds.

### Supplementary Information

Supplementary Information.

## Data Availability

All data generated and/ or analyzed during this study are available from the corresponding author on reasonable request.
