# Supplementary material for: Persistence of foliar applied and pre-storage seed-treated insecticides in rice and its processed products
Source: Sci Rep. 2024 Jan 29;14:2406. doi: 10.1038/s41598-024-53060-w (PMC10825215; doi:10.1038/s41598-024-53060-w)
Supplement: Supplementary file 1 — Supplementary Information. [file 41598_2024_53060_MOESM1_ESM.docx]

**Supplementary material**

**Title: Persistence of foliar applied and pre-storage seed-treated insecticides in rice and its processed products**

A. Suganthi^a *^, R. Vigneshwari^b^, N.Sathiah^a^, M. Senthil Kumar^c^, A.P.Sivamurugan^d^, P. Thangachamy^a^, S.S. Ilango^a^, E. Madhu Sudhanan^a^, P. Karthik^a^  and M.Shanthi^e^

^a^Department of Agricultural Entomology, Centre for Plant Protection Studies, Tamil Nadu Agricultural University, Coimbatore, 641 003, Tamil Nadu, India

^b^Department of Seed Science and Technology, Tamil Nadu Agricultural University, Coimbatore, 641 003, Tamil Nadu, India

^c^Krishi Vigyan Kendra, Thiruvallur, Tamil Nadu Agricultural University, Coimbatore, 641 003, Tamil Nadu, India

^d^Water Technology Centre, Tamil Nadu Agricultural University, Coimbatore, 641 003, Tamil Nadu, India

^e^ Centre for Plant Protection Studies, Tamil Nadu Agricultural University, Coimbatore, 641 003, Tamil Nadu, India

* Corresponding author E-mail address: [suganthi.a@tnau.ac.in](mailto:suganthi.a@tnau.ac.in);

Pages 5

Figure 1

**Materials and methods**

Validation datasets were collected following SANTE guidelines (SANTE 2019). Parameters, including selectivity, linearity, the limit of detection (LOD), the limit of quantification, recovery (accuracy), and repeatability (precision), were evaluated. All analyses were conducted using organically grown rice samples obtained from the Department of Sustainable Organic Agriculture, Tamil Nadu Agricultural University (TNAU), Coimbatore.

**a. Specificity:** The specificity of the analytes was confirmed by matching the retention time of the standard and then comparing it with the control blank, ensuring that the peak obtained is specifically from the analyte and not from the matrix. The analyte solution was injected six times at one concentration.

**b. Linearity studies:** The linearity of detector response for the analyte was evaluated by injecting a range of working standard solutions in the concentration levels from 0.005 to 0.5 µg mL^-1^ in LC-MS/MS with three replicate injections per concentration. Linear relationships among peak area signals and the corresponding concentrations were observed.

To measure the detection limit, 5 samples of concentrations that were spiked at 0.025 μg g^-1^ were analyzed and the [standard deviation](https://en.wikipedia.org/wiki/Standard_deviation) was calculated. The LOD was determined from the standard deviation associated with the measurement of the pesticides and student t-test value (n-1 degrees of freedom) and LOQ was calculated as 3 x LOD.

**c. Matrix effects:** The matrix effects of different rice matrices were assessed by comparing the slopes of matrix-matched calibration curve with the slopes of the solvent calibration curve.

ME = ((Slope of matrix matched standard curve / Slope of neat standard curve) - 1) x 100

A matrix effect of 0 to 20 per cent is considered as less effect, 20 to 50 per cent is medium effect and greater than 50% is considered strong effect.

**d. Recovery and Precision:** The methodological accuracy was assessed through the five-level spiking samples while the precision was studied in terms of repeatability and intra-day precision. These were obtained with recovery studies carried out by samples spiked at levels of 0.025, 0.0625, 0.125, 0.1875 and 0.250 µg g^-1^ following five replications for grain, bran and leaf matrix. Matrix match standards were used for quantification. With every batch of samples, blank samples were injected. The spiked samples were equilibrated and processed by adopting the above-said extraction and clean-up procedure. The repeatability of the method was evaluated through the relative standard deviation (RSD %).

**Results:**

**Method performance**

Five attributes of the extraction and analysis methods were validated: Linearity, accuracy, precision (% RSD), the limit of detection (LOD) and limit of quantification (LOQ). The obtained recovery data and validation parameters are presented in Fig S1.

The described method exhibited excellent performance characteristics across all tested matrices. Robust linearity results were achieved with regression coefficients exceeding 0.99 for both thiamethoxam and chlorantraniliprole. In the specificity study, the % RSD of retention time was below 2%, and the % RSD of the area was less than 10%, meeting acceptable criteria.

The experimentally determined limit of detection and quantification for rice grain, bran, and straw were 0.008 and 0.025 µg g^-1^, respectively. The obtained LOQs were comfortably above the CODEX MRL of 0.4 mg kg^-1^ for rice grain and 30 mg kg^-1^ for straw regarding chlorantraniliprole residues. For thiamethoxam residues in rice grain, where CODEX MRL is unavailable, the Japanese MRL of 0.3 mg kg^-1^ for brown rice (JFCRF, 2022) was considered.

The removal of interferences during the extraction and cleanup steps of sample preparation proved sufficient for reliable analyte identification and subsequent quantification. Rice whole grain, bran, and leaves were selected as representative commodities for method validation studies. A medium matrix effect (20 to 50%) was observed for different rice matrices, except for chlorantraniliprole in the bran matrix (17.52%). To compensate for the matrix effect and prevent any overestimation or underestimation of residues, matrix-matched calibration of respective matrices was employed for quantifying residues.

The recovery ranged from 70% to 120% for whole grain and bran matrices, while it was 61.63% to 87.47% for rice leaf samples. Notably, the recovery in rice leaves fell below 70%. This lower recovery in rice leaves can be attributed to their higher hemicellulose content and lower levels of cellulose and lignin, which may have interfered with the extraction of residues. Despite this, the relative standard deviations (RSDs) for the five spiked levels were consistently lower than 20% for all matrices. The efficiency of extraction strongly depends on the solvent used, the type of sample, and the chemical properties of the pesticides. Acetonitrile emerged as an efficient solvent for all rice samples, and the cleanup sorbents, namely PSA and anhydrous MgSO_4_, were found to be effective. Satisfactory parameters were achieved with a sample weight of 5 g for rice grain and bran matrices and 2 g for leaf samples.

The developed method offers several advantages, including low salt consumption, an overall low cost of analysis, and high recoveries across various rice matrices. In a prior study, Telo et al. (2017) also employed acetonitrile for extracting rice grain and hull samples. However, in their cleanup step, they used 500 mg of C18, whereas in the present method, only 100 mg of PSA was utilized. They reported a LOD of 0.01 mg kg^-1^ for rice grain and 0.02 mg kg^-1^ for hull.

**Summary:**

The method validation for residue analysis of thiamethoxam and chlorantraniliprole was conducted in accordance with the criteria outlined in the SANTE guidelines. The achieved results met the required standards, with acceptable recovery falling within the range of 70% to 120% and relative standard deviations consistently below 20% for all tested rice matrices.

The determined limits of quantification (LOQs) were comfortably higher than the specified CODEX Maximum Residue Limits (MRLs). For chlorantraniliprole residues, the LOQs surpassed the CODEX MRLs of 0.4 mg kg^-1^ for rice grain and 30 mg kg-1 for straw. Similarly, for thiamethoxam residues in brown rice, the LOQs exceeded the Japanese MRL of 0.3 mg kg^-1^.

**Fig. S1** Average recovery (%), RSDs of thiamethoxam and chlorantraniliprole from rice matrices
